# Reevaluation of ATR signaling in primary resting chronic lymphocytic leukemia cells: evidence for pro-survival or pro-apoptotic function

**DOI:** 10.18632/oncotarget.18144

**Published:** 2017-05-24

**Authors:** Maxime Beyaert, Eliza Starczewska, Ana Cristina González Pérez, Nicolas Vanlangendonck, Pascale Saussoy, Gaëlle Tilman, Anne De Leener, Marie-Christiane Vekemans, Eric Van Den Neste, Françoise Bontemps

**Affiliations:** ^1^ de Duve Institute, Université catholique de Louvain, B-1200 Brussels, Belgium; ^2^ Department of Hematology, Cliniques universitaires Saint-Luc, Université catholique de Louvain, B-1200 Brussels, Belgium; ^3^ Service de Biologie clinique, Cliniques universitaires Saint-Luc, Université catholique de Louvain, B-1200 Brussels, Belgium; ^4^ Center for Human Genetic, Cliniques universitaires Saint-Luc, Université catholique de Louvain, B-1200 Brussels, Belgium

**Keywords:** ATR, CLL, fludarabine, UV-C, p53

## Abstract

ATM, primarily activated by DNA double-strand breaks, and ATR, activated by single-stranded DNA, are master regulators of the cellular response to DNA damage. In primary chronic lymphocytic leukemia (CLL) cells, ATR signaling is considered to be switched off due to ATR downregulation. Here, we hypothesized that ATR, though expressed at low protein level, could play a role in primary resting CLL cells after genotoxic stress. By investigating the response of CLL cells to UV-C irradiation, a prototypical activator of ATR, we could detect phosphorylation of ATR at Thr-1989, a marker for ATR activation, and also observed that selective ATR inhibitors markedly decreased UV-C-induced phosphorylation of ATR targets, including H2AX and p53. Similar results were obtained with the purine analogs fludarabine and cladribine that were also shown to activate ATR and induce ATR-dependent phosphorylation of H2AX and p53. In addition, ATR inhibition was found to sensitize primary CLL cells to UV-C by decreasing DNA repair synthesis. Conversely, ATR inhibition rescued CLL cells against purine analogs by reducing expression of the pro-apoptotic genes *PUMA* and *BAX*. Collectively, our study indicates that ATR signaling can be activated in resting CLL cells and play a pro-survival or pro-apoptotic role, depending on the genotoxic context.

## INTRODUCTION

Chronic lymphocytic leukemia (CLL) is characterized by an extremely heterogeneous clinical course, remaining stable for many years in some patients, while others have an aggressive form of the disease that is refractory to chemotherapy [1, 2]. Adverse CLL is often associated with loss of functional p53, resulting in defective apoptosis in response to genotoxic therapy and shortened survival times [3, 4]. Mechanistically, p53 dysfunction can result from *TP53* deletion (17p deletion) and/or mutations, but also from impaired activation by upstream DNA damage-activated kinase(s) belonging to the phosphoinositide 3-kinase (PI3K)-like family of protein kinases [5]. Among them, ATM (ataxia telangiectasia mutated), a kinase activated in response to DNA double-strand breaks, is assumed to play a central role in the control of p53 function in CLL. Consistent with this, CLL patients with *ATM* abnormalities (11q deletion and/or *ATM* mutations) display higher refractoriness to DNA-damaging drugs, such as alkylating agents and fludarabine [6–8]. However, it has been reported that primary CLL cells with pharmacologically or naturally inactivated ATM could exhibit p53 phosphorylation in response to fludarabine, suggesting that other signaling kinases might be involved in p53 activation [9]. The possibility that the closely related kinase ATR (ataxia telangiectasia and Rad3-related), which is activated by DNA lesions that induce the formation of single-strand DNA [10], could play a role in p53 signaling in CLL was previously ruled out. Indeed, the p53 pathway was found not to be activated in response to UV-C irradiation, a well-established activator of ATR. This was explained by strong downregulation of ATR in resting CLL cells in comparison with proliferating cells [11].

While it is largely accepted that ATR does not play a role in the cellular response to DNA damage in resting CLL cells, recent data we obtained about the mechanism of activation of deoxycytidine kinase (dCK) led us to reconsider this view. dCK is a rate-limiting enzyme in deoxyribonucleoside salvage and nucleoside analog activation [12, 13], which is activated in response to genotoxic stress through Ser-74 phosphorylation [14, 15]. Whereas ATM was identified as the kinase that phosphorylates Ser-74 and activates dCK in response to ionizing radiation (IR) [16, 17], we demonstrated using various cell lines that ATR was responsible for dCK activation after UV-C exposure [18]. However, activation of dCK by UV-C light was observed not only in normal or cancer cell lines, but also in primary resting CLL lymphocytes [15, 19], which suggested that ATR might be functional in these cells. The present study was initiated to explore this hypothesis. We investigated whether ATR could be activated by genotoxic agents, namely UV-C and chemotherapeutic purine analogs, and used highly specific ATR inhibitors to evaluate the biological consequences of this potential activation. We provide evidence that ATR, although present at low protein level, can play a role in DNA damage response (DDR) in resting CLL cells, exerting pro-survival or pro-apoptotic function depending on the genotoxic event.

## RESULTS

### ATR protein can be detected in primary resting CLL cells

**Figure 1:**
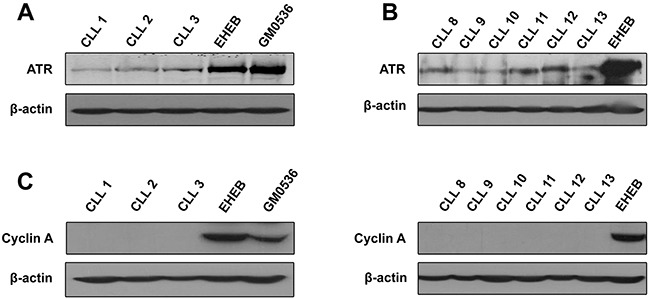
ATR protein level in primary resting CLL cells as compared with proliferating cells Same amounts of lysates obtained from different CLL samples or from EHEB and GM0536 cells were analyzed by immunoblotting for ATR, using a Santa Cruz **(A)** or a Cell Signaling antibody **(B).** Cyclin A **(C)** was analyzed to verify the quiescent state of CLL cells. β-Actin was used as a loading control.

**Figure 2:**
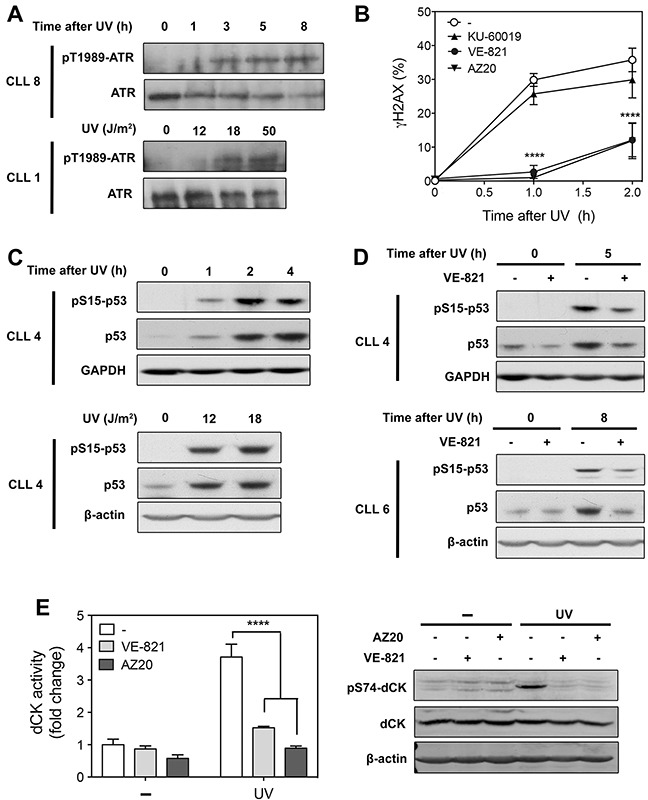
Analysis of ATR signaling in primary resting CLL cells after UV-C irradiation **(A)** Time- and dose-dependent activation of ATR by UV-C. CLL cells from 2 different patients were irradiated with UV-C (18 J/m^2^) and incubated for the indicated times (upper panel), or irradiated with increasing UV-C doses and incubated for 5 h (lower panel). Cell lysates were probed for phospho-ATR(Thr-1989) and ATR protein level. **(B)** Effect of ATR and ATM inhibitors on UV-C-induced γH2AX accumulation. CLL cells were preincubated with or without 10 μM VE-821, 1 μM AZ20 or 10 μM KU-60019 before UV-C irradiation (18 J/m^2^). At the indicated times, phosphorylation of H2AX (γH2AX) was analyzed by flow cytometry and expressed as fold increase over untreated cells. Results are means ± SEM of 5 independent experiments. **(C)** Time- and dose-dependent activation of p53 after UV-C exposure. CLL cells were irradiated with UV-C (18 J/m^2^) and incubated for the indicated times (upper panel), or irradiated with UV-C at 12 and 18 J/m^2^ and incubated for 5 h (lower panel). Cell lysates were probed for phospho-p53(Ser-15) and p53 protein level. GAPDH or β-actin were used as loading controls. **(D)** Effect of VE-821 on UV-C-induced p53 activation. CLL cells from two different patients were preincubated with 10 μM VE-821 before UV-C irradiation (18 J/m^2^). Phosphorylation of p53 at Ser-15 and p53 protein level were analyzed by Western blot 5 h (upper panel) or 8 h (lower panel) after irradiation. **(E)** Effect of ATR inhibitors on dCK activation and phosphorylation induced by UV-C. CLL cells were preincubated with or without 10 μM VE-821 or 1 μM AZ20 before UV-C irradiation (18 J/m^2^). dCK activity (n = 3, left panel) and phospho-dCK(Ser-74) (right panel) were analyzed 30 min after irradiation. Basal dCK activity was 10.1 ± 1.7 pmol/min/mg protein (n = 3). Significance relative to the absence of ATR inhibitors (panels B and E): ****P < 0.0001.

**Figure 3:**
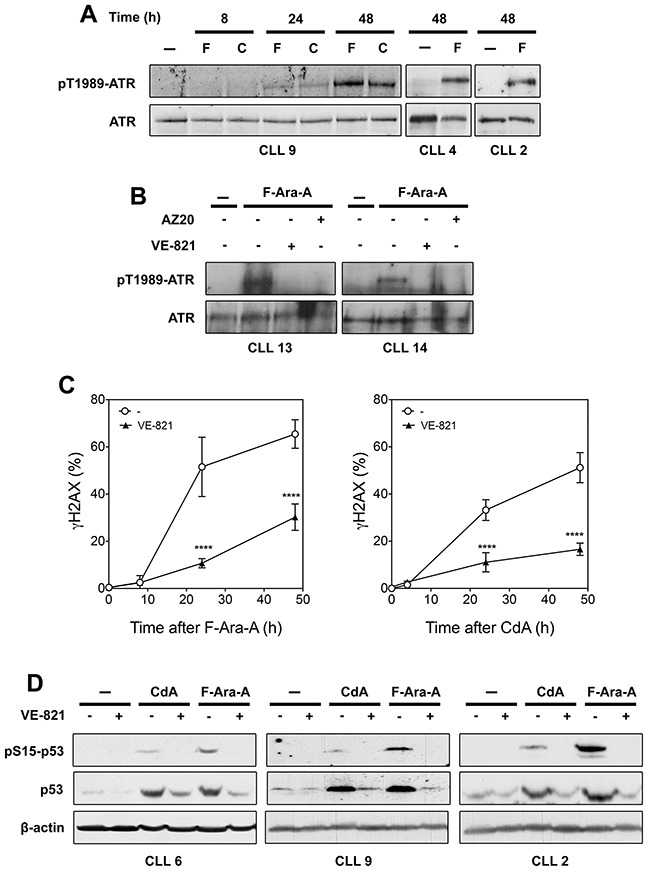
Analysis of ATR signaling in primary resting CLL cells after treatment with fludarabine (F-Ara-A) or cladribine (CdA) **(A)** Activation of ATR by purine analogs. CLL cells were incubated with 3 μM F-Ara-A (F) or 1 μM CdA (C) for various times, or with or without 3 μM F-Ara-A for 48 h. Phospho-ATR(Thr-1989) and ATR protein level were analyzed by Western blot. **(B)** Effect of ATR inhibitors on fludarabine-induced ATR phosphorylation. CLL cells were incubated for 48 h with or without 3 μM F-Ara-A in the presence or absence of 10 μM VE-821 or 1 μM AZ20, followed by analysis of ATR phosphorylation by Western blot. **(C)** Effect of VE-821 on γH2AX accumulation induced by F-Ara-A (left panel) or CdA (right panel). CLL cells were preincubated with or without 10 μM VE-821 before addition of 3 μM F-Ara-A or 1 μM CdA. At the indicated times, phosphorylation of H2AX (γH2AX) was analyzed by flow cytometry and expressed as fold-increase over untreated cells. Results are means ± SEM of 4 independent experiments. Significance relative to the absence of VE-821: ****P < 0.0001. **(D)** Effect of VE-821 on p53 activation induced by fludarabine or cladribine. CLL cells from three different patients were preincubated with 10 μM VE-821 before addition of 1 μM CdA or 3 μM F-Ara-A. Phospho-p53(Ser-15) and p53 protein level were analyzed by Western blot after 24 h of incubation. β-Actin were used as a loading control.

As previously reported [11, 20], we found that signal for ATR protein was not easily detectable in resting CLL cells, regardless of the ATR antibody used (Figure 1A and 1B), and required extended exposure time of the blot. Nevertheless, even if a certain inter-patient variability was observed, presence of ATR was demonstrable in all CLL samples analyzed (see also in Figures 2 and 3). Comparison with the CLL cell line EHEB or the lymphoblastoid cell line GM0536 confirmed that ATR protein expression was markedly lower in quiescent CLL cells than in proliferating cells [11, 20]. We verified by flow cytometry that primary peripheral blood CLL cells were out of the cell cycle *in vitro* (not shown) and did not express the protein cyclin A, a marker for cell proliferation, in contrast with EHEB and GM0536 cells (Figure 1C).

### UV-C irradiation activates ATR in CLL cells and induces phosphorylation of ATR substrates

We first investigated whether ATR could be activated in CLL cells in response to an established ATR activator, namely UV-C light, which can be done by analyzing ATR autophosphorylation at Thr-1989 [21]. We observed that ATR became indeed phosphorylated after UV-C irradiation in a time- and dose-dependent manner (Figure 2A).

To confirm ATR activation in response to UV-C, we examined phosphorylation of different ATR targets. Phosphorylation of Chk1, commonly used as readout of ATR activation [22], could not be analyzed given that the Chk1 protein was undetectable in primary CLL cells, as observed by others in quiescent or G1-phase cells [20, 23]. Besides Chk1, the histone variant H2AX, which is rapidly phosphorylated at Ser-139 by ATM in response to ionizing radiation (IR), has been shown to be an ATR target upon UV-C exposure in dividing as in quiescent cells [24–27]. In line with this, we observed that phosphorylation of H2AX (γH2AX) induced by UV-C in CLL cells was completely prevented for up to 60 min by two selective ATR inhibitors, VE-821 and AZ20 [28, 29], used at 10 and 1 μM, respectively (Figure 2B). In contrast, γH2AX accumulation was minimally affected by 10 μM KU-60019, a specific ATM inhibitor [30]. Importantly, we confirmed that VE-821 and AZ20 did not inhibit the accumulation of γH2AX induced by IR, whereas KU-60019 strongly reduced it (Supplementary Figure 1). These data established that VE-821 and AZ20, at concentrations used in our experiments, did not influence ATM activity and could be used as tools to investigate ATR signaling, as has been done in number of studies [28, 31–35].

A major argument against active ATR signaling in primary CLL cells was the previous finding that p53 did not accumulate in resting CLL cells after UV-C irradiation [11]. Nevertheless, perhaps due to higher sensitivity of Western blot itself, we were able to detect phosphorylation of p53 at Ser-15 as well as accumulation of p53 protein after UV-C irradiation, which were time- and dose-dependent (Figure 2C). Moreover, we found that p53 activation was reduced in the presence of VE-821 (Figure 2D), indicating that ATR contributes to this activation.

Finally, we examined whether activation of dCK, which had previously been observed in quiescent CLL cells after UV-C irradiation [15], was actually mediated by the kinase ATR. As expected, increase of dCK activity (Figure 2E, left panel) as well as dCK phosphorylation at Ser-74 induced by UV-C (Figure 2E, right panel) were almost completely suppressed by the ATR inhibitors VE-821 and AZ20. These results mirrored exactly those previously obtained in cell lines, in which transfection of ATR siRNA had confirmed the role of ATR in dCK activation in response to UV-C [18].

### As UV-C, fludarabine and cladribine activate ATR in CLL cells and induce phosphorylation of ATR substrates

While UV-C light is a well-established ATR activator, whether chemotherapeutic purine analogs could activate ATR had been little investigated, and especially not in primary CLL cells in which the ATR pathway was thought to be suppressed [11]. Nevertheless, we had previously observed that cladribine induced phosphorylation of the ATR target Chk1 in lymphoblastoid and leukemic cell lines [18], which encouraged us to analyze whether ATR was activated in response to purine analogs in CLL cells. As displayed in Figure 3A, phosphorylation of ATR at Thr-1989 was not detectable after 8 h of incubation with 3 μM fludarabine or 1 μM cladribine, but became apparent after 24 and 48 h. Additional analyses showed that ATR phosphorylation induced by fludarabine could be prevented by the ATR inhibitors VE-821 and AZ20, corroborating that ATR undergoes autophosphosphorylation and thus activation in response to this chemotherapeutic drug (Figure 3B). Activation of ATR by purine analogs was markedly slower than with UV-C, which can most probably be related to the time required for accumulation of sufficient genotoxic damage. Indeed, incorporation of purine analogs into DNA only arises through background DNA repair synthesis in resting cells [36].

Induction of γH2AX in response to purine analogs in primary CLL cells has been reported in several studies [37–39]. We show here that γH2AX accumulation, whether induced by fludarabine (Figure 3C, left panel) or cladribine (Figure 3C, right panel), was significantly reduced by the ATR inhibitor VE-821. Activation of p53 in response to purine analogs, which is well documented in primary CLL cells [36], was also found to be inhibited by VE-821 (Figure 3D): both p53 phosphorylation and p53 accumulation were indeed strongly reduced by the ATR inhibitor, indicating that ATR plays an important role in stabilizing p53 after purine analog treatment. That ATR could be involved in p53 induction by fludarabine or cladribine was corroborated by experiments performed in the CLL cell line EHEB, in which depletion of ATR by siRNA paralleled the inhibitory effect of VE-821 on p53 phosphorylation and accumulation (Supplementary Figure 2). Collectively, these data show that chemotherapeutic purine analogs activate ATR signaling in primary resting CLL cells as in cell lines.

### ATR inhibitors enhance UV-C-induced cytotoxicity, but protect CLL cells against purine analogs

Since ATR was activated in response to UV-C irradiation or purine analogs in CLL cells and was able to phosphorylate targets, including p53, a key player of DDR, we reasoned that ATR could play a role in the cellular response to these DNA-damaging treatments. To address this question, we analyzed their cytotoxicity as well as activation of caspase-3, an indicator of apoptosis, in the presence or absence of ATR inhibitors.

**Figure 4:**
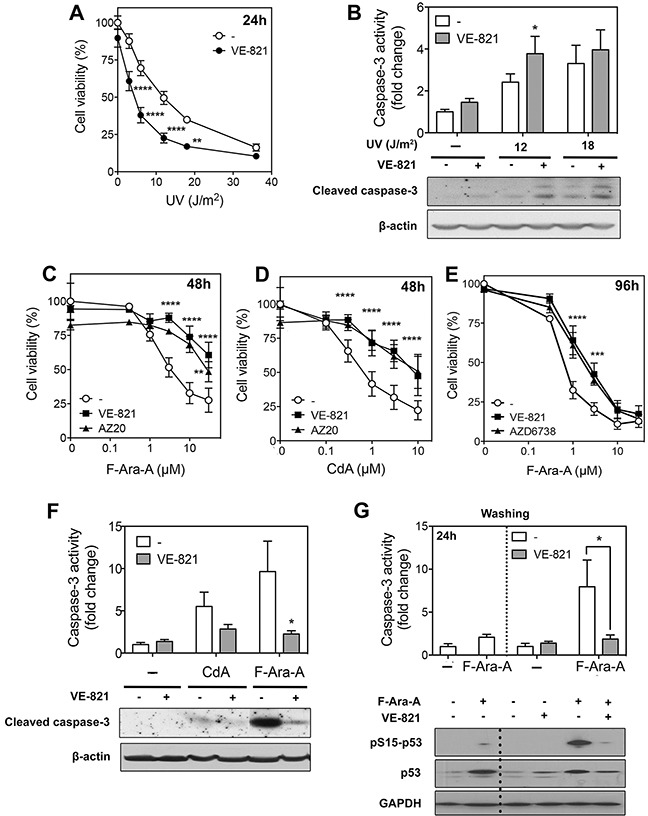
Effect of ATR inhibitors on cell death induced by UV-C irradiation or purine analogs in primary resting CLL cells **(A)** Effect of VE-821 on UV-C-induced cytoxicity. CLL cells were preincubated with or without 10 μM VE-821 and then exposed to increasing UV-C doses, as indicated. Cell viability was measured by the MTT assay after 24 h of incubation. Results are means ± SEM of 4 separate experiments. **(B)** Effect of VE-821 on caspase-3 activation induced by UV-C irradiation. CLL cells were preincubated with or without 10 μM VE-821 and then irradiated with UV-C at 12 or 18 J/m^2^. Caspase-3 activity was analyzed 8 h after irradiation by an enzymatic assay (n=3, upper panel) or by immunoblotting of its active cleaved form (lower panel). **(C-D)** Influence of ATR inhibitors on purine analog-induced cytotoxicity. CLL cells were incubated for 48 h with F-Ara-A (C) or CdA (D) at increasing concentrations in the absence or presence of VE-821 or AZ20. Cell viability was measured by the MTT assay. Results are means ± SEM of 4 separate experiments. **(E)** Effect of AZD6738 as compared to VE-821 on F-Ara-A-induced cytotoxicity. CLL cells were incubated for 96 h with F-Ara-A at increasing concentrations in the presence or absence of 10 μM VE-821 or 1 μM AZD6738. Cell viability was measured by the MTT assay. Results are means ± SEM of 3 separate experiments. **(F)** Effect of VE-821 on caspase-3 activation induced by CdA or F-Ara-A. CLL cells were preincubated with or without 10 μM VE-821 and incubated in the presence or absence of 1 μM CdA or 3 μM F-Ara-A. Caspase-3 activity was analyzed after 48 h by an enzymatic assay (n = 3, upper panel) or by immunoblotting of its active cleaved form (lower panel). **(G)** Effect of VE-821 when added 24 h after fludarabine. CLL cells were incubated for 24 h in the presence or absence of 3 μM F-Ara-A, washed from F-Ara-A, and resuspended in fresh medium containing, or not, 10 μM VE-821. Caspase-3 activity was measured just before washing and after additional 24 h, by an enzymatic assay (upper panel). Results are means ± SEM of 4 independent experiments. p53 phosphorylation and p53 protein level were also analyzed at the same incubation times by Western blot (lower panel). GAPDH was used as a loading control. The dotted lines in the upper and lower panels indicate washing of cells to remove F-Ara-A. Significance relative to the absence of ATR inhibitors: *P < 0.05; **P <0.01; ***P < 0.001; ****P < 0.0001.

The effect of ATR inhibition on UV-C cytotoxicity was analyzed 24 h after irradiation (Figure 4A). As already observed in quiescent cell models [40], VE-821 alone did not significantly affect CLL cell viability. However, it markedly enhanced UV-C cytotoxicity, particularly at low UV-C doses. In line with this, activation of caspase-3 was also more pronounced in the presence of VE-821 (Figure 4B, upper panel). Higher activation of caspase-3 in the presence of VE-821 was confirmed by analysis of the cleaved form of caspase-3 by Western blot (Figure 4B, lower panel).

The effect of ATR inhibition on purine analog cytotoxicity was first analyzed after a 48 h-incubation. In contrast with UV-C, we observed that the ATR inhibitor VE-821 as well as AZ20 did not increase, but conversely decreased the cytotoxicity of fludarabine (Figure 4C) or cladribine (Figure 4D). This protective effect of ATR inhibition was also recorded after a 96 h-incubation (Supplementary Figure 3 and Figure 4E). Moreover, the protective effect of VE-821 could be mimicked by using AZD6738 (1 μM) (Figure 4E), an ATR inhibitor recently entered into clinical trials, which was shown to induce synthetic lethality in *TP53*/*ATM*-defective CLL cells and sensitize them to several chemotherapeutic drugs, including fludarabine [20]. However, it should be noted that the latter study was conducted in proliferating DDR-defective CLL cells in which ATR inhibition leads to cell death by mitotic catastrophe [20]. Finally, consistent with the protective effect of VE-821 observed in cytotoxicity assays, we found that activation of caspase-3 in response to fludarabine or cladribine was significantly reduced in the presence of the ATR inhibitor (Figure 4F), indicating a decrease in apoptosis.

Given that the conversion of purine analogs into their active triphosphate form and their incorporation into DNA are a prerequisite for cytotoxicity [36], we verified whether these processes were not affected by ATR inhibition. We found that the metabolism of cladribine was not significantly modified by a 24 h-incubation in the presence of VE-821, while activation of fludarabine and its incorporation into DNA were reduced by about 30% (Supplementary Table 1). However, this inhibitory effect of VE-821 was no longer detected after 48 h. To verify whether the decrease in fludarabine metabolism observed after 24 h could play a substantial role in the decrease of fludarabine cytotoxicity induced by VE-821, we performed experiments in which DNA damage was induced before addition of the ATR inhibitor. CLL cells were thus incubated for 24 h in the presence or absence of 3 μM fludarabine, washed twice with fresh culture medium and then re-incubated for 24 h with or without VE-821. Caspase-3 activity clearly increased in cells that had been preincubated in the presence of fludarabine, while not in the presence of VE-821 (Figure 4G, upper panel), confirming the role of ATR in fludarabine-induced apoptosis. In parallel, p53 phosphorylation and accumulation were found to be reduced by VE-821 (Figure 4G, lower panel), corroborating the role of ATR in p53 induction by fludarabine. Therefore, the decrease of fludarabine cytotoxicity induced by VE-821 could not, or at least not entirely, be explained by a reduced activation of fludarabine. The reason why VE-821 slightly slowed down fludarabine activation is currently unclear. Inhibition of dCK should not be implicated because phosphorylation of dCK on Ser-74 has been shown to enhance dCK activity towards deoxycytidine and pyrimidine analogs, but not deoxyadenosine and purine analogs [41].

### VE-821 inhibits DNA repair synthesis induced by UV-C, while it decreases pro-apoptotic gene expression induced by purine analogs

**Figure 5:**
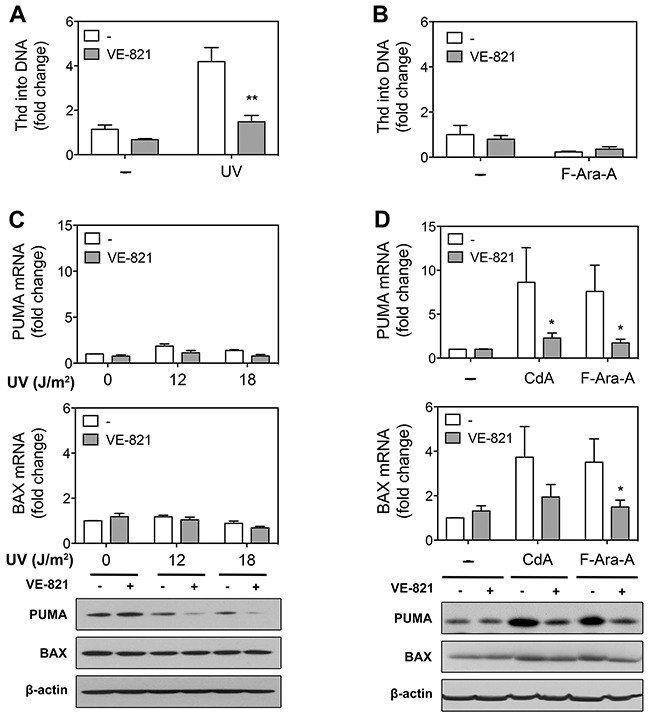
Effect of ATR inhibition on DNA repair synthesis and pro-apoptotic gene expression in primary resting CLL cells after treatment with UV-C or purine analogs **(A)** DNA repair synthesis after UV-C irradiation. After preincubation with or without 10 μM VE-821, CLL cells were, or not, UV-C-irradiated (18 J/M^2^) and incubated with labeled thymidine (Thd) for 3 h. Results are means ± SEM of 3 independent experiments. **(B)** DNA repair synthesis after treatment with F-Ara-A. After preincubation with or without 10 μM VE-821, CLL cells were incubated in the absence or presence of 3 μM F-Ara-A for 43 h, and then incubated with labeled thymidine for 5 h. Results are means ± SEM of 3 independent experiments. **(C)** PUMA and BAX expression after UV-C irradiation. After preincubation with or without 10 μM VE-821, CLL cells (n = 3) were UV-C irradiated (18 J/m^2^) and incubated for 8 h before analysis of PUMA and BAX expression by qPCR (upper and middle panels) and Western blot (lower panel). **(D)** PUMA and BAX expression after purine analogs. After preincubation with or without 10 μM VE-821, CLL cells (n = 3) were incubated with 1 μM CdA or 3 μM F-Ara-A for 24 h before analysis of PUMA and BAX expression by qPCR (upper and middle panel) and Western blot (lower panel). Significance relative to the absence of VE-821 (panels A, B and D): *P < 0.05; **P <0.01.

Next step was to unravel the mechanisms by which ATR inhibitors could either increase or decrease the cytotoxicity induced by DNA damage. Major roles of DDR are to promote DNA repair and cell survival or to induce apoptosis at high DNA damage, the decision between survival and death depending on several factors not yet entirely understood [42]. Since inhibition of ATR resulted in higher UV-C cytotoxicity, we speculated that a pro-survival function of ATR, such as DNA repair, could be inhibited. The major system to remove UV-induced DNA photolesions is the nucleotide excision repair (NER) pathway, which can be investigated in resting cells, such as normal lymphocytes and CLL cells, by analyzing thymidine incorporation into DNA in the presence of hydroxyurea [43]. Using this assay, we found that DNA repair synthesis elicited by UV-C light was indeed markedly reduced in the presence of VE-821 (Figure 5A). In contrast, DNA repair synthesis was not stimulated, but rather decreased after incubation with fludarabine, and was not modified by VE-821 (Figure 5B).

Regarding purine analogs, ATR inhibition resulted in a decrease of their cytotoxicity, suggesting that ATR exerts a pro-apoptotic function in this condition. It is well established that apoptotic cell death induced by purine analogs involves p53-dependent and -independent mechanisms [36] and that p53-dependent apoptosis in response to fludarabine is mediated at least partly by induction of pro-apoptotic genes such as *PUMA* and *BAX* [44, 45]. Therefore, we sought to investigate the effect of ATR inhibition on PUMA and BAX expression in response to cladribine and fludarabine (Figure 5D). It appeared that up-regulation of PUMA and BAX mRNA was significantly reduced by VE-821. Induction of PUMA protein by cladribine and fludarabine was similarly counteracted by the ATR inhibitor. As for BAX, its protein level was only modestly increased by purine analogs, so that the effect of VE-821 was marginal. In comparison, BAX and PUMA expression was barely or not increased after UV-C irradiation (Figure 5C), which indicated that death of CLL cells induced by UV-C irradiation was not mediated by increased expression of pro-apoptotic genes and therefore would be p53-independent, although UV-C also induced p53 upregulation.

**Figure 6:**
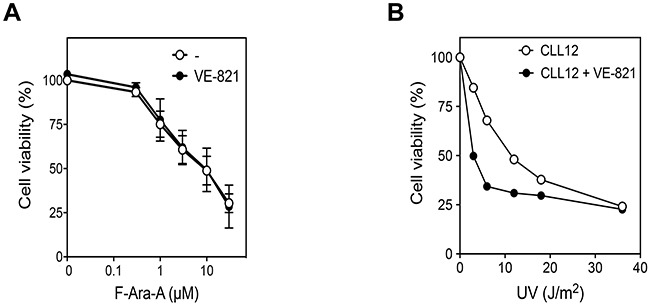
Effect of ATR inhibition on fludarabine- and UV-C -induced cytotoxicity in p53-defective CLL cells **(A)** 17p-deleted CLL samples (CLL12, 23 and 40), containing ≥ 71 % of cells with deletion, were incubated for 96 h with increasing concentration of F-Ara-A in the presence or absence of 10 μM VE-821. **(B)** One of these samples (CLL12) was also irradiated with increasing UV-C doses and incubated for 24 h in the presence or absence of 10 μM VE-821. Cell survival was measured by the MTT assay.

Interestingly, we could investigate the effect of VE-821 on fludarabine cytotoxicity in three del(17p) CLL samples (containing ≥ 71 % of cells with 17p deletion) (Figure 6A). We observed that fludarabine cytotoxicity, which was ∼ 10-fold lower than in non-deleted cells, was not affected by the ATR inhibitor (Figure 6A). In contrast, in one of these samples (CLL12), which besides fludarabine could also be treated by UV-C, VE-821 was still able to increase UV-C-cytotoxicity (Figure 6B) similarly to what was observed in non-deleted samples (Figure 4A). These results support the hypothesis that the pro-survival function of ATR, such as observed after UV-C, would be p53-independent in contrast to its pro-apoptotic function, such as observed after purine analogs.

## DISCUSSION

Even if clinically aggressive and refractory CLL is most often associated with high proliferation rate in secondary lymphoid organs [46] and current therapies predominantly aim to eradicate dividing CLL cells, almost all CLL cells of the peripheral blood are cell cycle-arrested [47] and deserve thus to be thoroughly characterized. Particularly, a detailed understanding of their response to genotoxic drugs, which are still a cornerstone in the treatment of CLL, is fundamental for development of rational therapy. Indeed, the DDR plays an important role in the effectiveness of DNA-damaging drugs, leading to apoptotic cell death by inducing p53 stabilization, but also compromising their efficacy by facilitating DNA repair [48]. While ATM was recognized as a crucial regulator of DDR in CLL, ATR was thought to be switched off in resting CLL cells [11] in contrast with proliferating CLL cells in which ATR signaling was clearly demonstrated [20]. The present study indicates for the first time, to our knowledge, that ATR can be activated in response to DNA damage in primary resting CLL cells and is involved in the cellular response to genotoxic stress. Remarkably, evidence in favor of this statement was obtained in all CLL samples analyzed in this study.

Activation of ATR in response to UV-C as well as purine analogs was demonstrated by ATR autophosphorylation at Thr-1989, a direct readout for ATR activation [21], and by the fact that phosphorylation of recognized ATR targets, namely H2AX, p53 and dCK, was totally or partially reduced by two selective ATR inhibitors, proven to be not inhibitory for other PI3K-like kinases, including ATM, at concentrations used in our study [21, 28, 35, 49]. Nonetheless, it remains intriguing that ATR can display activity in CLL cells despite a low protein level. Similarly, ATR-dependent phosphorylation of H2AX in response to UV-C was recently evidenced in non-replicating normal human peripheral blood mononuclear cells (PBMCs), which are also characterized by a weak ATR activity [26]. These observations could possibly be related to the finding that a 90% depletion of ATR was not deleterious in a mouse model [50], suggesting that ATR is largely overwhelming in replicating cells.

Interest for development of ATR inhibitors arises from their capacity to display selective cytotoxicity, as single agents or in combination with genotoxic chemotherapies, in tumor cells harboring p53 or ATM defects [51]. However, impact of ATR inhibition has especially been investigated in replicating DDR-defective cells in which ATR is critical for cell survival [52, 53]. We show here that ATR inhibitors can also influence the ultimate fate of resting CLL cells with no DDR defect when they are subjected to DNA damage. Unexpectedly, we found that inhibition of ATR sensitized quiescent CLL cells to UV-C irradiation, but decreased cytotoxicity and apoptotic cell death induced by purine analogs, showing that ATR can exert, within the same cell, a pro-survival or pro-apoptotic function depending on the genotoxic stress.

ATR inhibition resulted in a decrease of UV-C induced DNA repair synthesis, suggesting that ATR plays a pro-survival role in response to UV-C in CLL cells by promoting DNA repair, most likely by regulating NER [54–56]. On the other hand, ATR inhibition markedly reduced upregulation of PUMA and BAX expression induced by cladribine and fludarabine, suggesting that activation of ATR in response to purine analogs contributes to cell death by promoting expression of pro-apoptotic proteins. As PUMA and BAX induction in response to fludarabine is p53-dependent in CLL [44], the pro-apoptotic function of ATR in this context is most probably explained by its ability to induce p53 activation. This hypothesis is corroborated by our observation that fludarabine cytotoxicity in 17p-deleted CLL samples was not modified by inhibiting ATR.

The reason why p53 activation induced in CLL cells by UV-C irradiation did not lead to upregulation of pro-apoptotic proteins remains to be elucidated. Nevertheless, it is well known that the function of p53 is modulated by a variety of signals, including numerous post-translational modifications, so that induction of similar levels of p53 can elicit different responses [42, 57]. UV-C-induced apoptosis appears thus to be predominantly p53-independent in CLL cells, as observed in other cell types [40, 58–60]. Also, the pro-survival function of ATR after UV-C is likely p53-independent, as 17p-deleted cells were still sensitized to UV-C by ATR inhibition. It should be noted that ATR function after UV-C could depend on the cell type as ATR inhibition was found to be protective for non-cycling human keratinocytes exposed to UV-C light [40].

While activation of ATM in response to fludarabine has already been reported in primary CLL cells [39] as in B-cell lines [61], activation of ATR by purine analogs in CLL or other cells has never been described. Our study thus provides a new insight into the mechanism of action of these genotoxic drugs and indicates that ATR plays an important role in their efficiency. Nevertheless, further work is needed to determine the respective roles of ATR and ATM in the cellular response to purine analogs. Whether ATR is implicated in the mechanism of action of other therapies in CLL would be also worth investigating. Finally, although mutations in ATR are rare [10] and have not yet been investigated or identified in CLL to our knowledge, they might be worth considering in the future.

In conclusion, ATR emerges from this study as an important player of the response to DNA damage in resting CLL cells, which is of biological relevance as almost all peripheral blood CLL cells are non-proliferating cells. Regarding fludarabine, which is still a mainstay in CLL treatment, ATR appears to contribute to its efficacy, at least in p53-proficient CLL cells. Our findings can be also of interest regarding use of ATR inhibitors, which are promising treatment option for CLL patients with *ATM/TP53* defects [20], corroborating that use of these molecules has to be strictly kept for patients with a high proportion of *TP53/ATM-*defective subclones. Indeed, ATR inhibitors in combination with chemotherapy might protect resting cells that would not be *TP53/ATM*-defective, leading to a decrease of the overall response to treatment.

## MATERIALS AND METHODS

### Drugs and reagents

Fludarabine (2-fluoro-arabinosyladenine, F-Ara-A) was purchased from Sigma-Aldrich (St Louis, MO, USA). Cladribine (2-chloro-2′-deoxyadenosine, CdA) was supplied by Prof. J. Marchand (Laboratory of Organic Chemistry, Université catholique de Louvain, Belgium). [8-^3^H]-Cladribine (22 Ci/mmol), [8-^3^H]-fludarabine (11.9 Ci/mmol), [^3^H-methyl]-thymidine (84 Ci/mmol) and [5-^3^H]-deoxycytidine (20 Ci/mmol) were from Moravek Biochemicals (Brea, CA, USA). KU-60019, VE-821, AZD6738 and AZ20 were from Bio-Connect (Huissen, The Netherlands). Ac-DEVD-AMC (Ac-Asp-Glu-Val-Asp-AMC) and AMC (7-amino-4-methyl-coumarin) were from Alexis Biochemicals (San Diego, CA, USA). Other chemicals were from Sigma–Aldrich, Merck Biosciences (Gibbstown, NJ, USA) or Bio-Rad (Hercules, CA, USA) Laboratories.

### Isolation and incubation of primary CLL cells

PBMCs were obtained from 40 different CLL patients who had provided informed consent following protocol approval by the Ethics Committee of the Cliniques universitaires Saint-Luc (Brussels, Belgium). This study was conducted in accordance with the Declaration of Helsinki. All patients had an established diagnosis by standard morphological and immunological criteria [62], were free of any anticancer treatment for at least 3 months and had lymphocytes ≥ 30 × 10^9^/L. Patient characteristics are summarized in Supplementary Table 2. Conventional karyotype and interphase fluorescence *in situ* hybridization (FISH) analysis were performed as previously described [63]. PBMCs (> 90% CLL cells, as confirmed by flow cytometry analysis) were isolated by density-gradient centrifugation using Ficoll-Paque Plus (GE Healthcare Life Sciences, Diegem, Belgium). Cell cycle phase distribution of CLL cells was evaluated as reported before, using the BD CycleTEST PLUS DNA Reagent Kit (BD Biosciences, Franklin Lakes, NJ, USA) [43].

Freshly isolated or thawed CLL cells (10 × 10^6^ cells/ml) were cultured in RPMI-1640 medium with Glutamax, supplemented with 10% fetal calf serum and 1% penicillin-streptomycin, at 37°C in an atmosphere of 5% CO_2_ in air [64]. When used, inhibitors were added to CLL cells one hour before treatment. VE-821 was used at 10 μM, a concentration that efficiently inhibited ATR in cell lines as well as in human PBMCs [18, 26], while AZ20 and AZD6738 were used at 1 μM as in previous studies [20, 65]. The ATM inhibitor KU-60019 was used at 10 μM as before [18]. Exposure of CLL cells to UV-C or ionizing radiation (IR) was performed using an UVGL-25 lamp delivering its bulk at 254 nM or a ^137^Cs source at a dose rate of 2.43 Gy/min, respectively [18].

### Cell lines

The leukemic cell line EHEB (chronic B-cell leukemia), obtained from DSMZ-German Collection of Microorganisms and Cell culture (Braunschweig, Germany) and the lymphoblastoid cell line GM0536, obtained from the NIGMS Human Mutant Cell Repository (Camden, NJ, USA) were cultured as CLL cells, except that antibiotics were omitted.

### Western blot analysis

Cell protein extracts were obtained as previously described [14]. Samples (30-150 μg of protein) were resolved by 7% (for ATR) or 12% (for other proteins) dodecyl sulfate-polyacrylamide gel electrophoresis [18]. Proteins were visualized using the Odyssey infrared Imaging System from LI-COR Biosciences (Nebraska, NE, USA) or the Clarity Western ECL Substrate (Bio-Rad) and ECL chemiluminescence film (Fujifilm). Glyceraldehyde-3-phospho dehydrogenase or β-actin were used as loading controls. Adobe Photoshop CS6 was used for image processing.

Antibodies used in this study were: anti-p53 (#sc126) and anti-ATR (#sc1887) from Santa Cruz Biotechnology (Santa Cruz, CA, USA); anti-ATR (#2790), anti-p53-pS15 (#9284), anti-caspase-3 (#9665), anti-Chk1 (#2360), anti-Chk1-pS317 (#2344) anti PUMA (#4976) and anti-BAX (#5023) from Cell Signaling Technologies (Beverly, MA, USA); anti-ATR-pT1989 (GTX128145) from GeneTex (Irvine, CA, USA); Alexa Fluor 488 Mouse anti-H2AX (pS139) from BD Biosciences (Franklin Lakes, NJ, USA); anti-β-actin and anti-glyceraldehyde-3-phospho dehydrogenase (GAPDH) from Sigma-Aldrich; anti-dCK-pS74 and anti-dCK were generated as previously described [14]. Secondary antibodies were purchased from Sigma–Aldrich (anti-rabbit and anti-mouse IG conjugated to horseradish peroxidase) or from Westburg (Leusden, The Netherlands) (IRDye 800CW donkey anti-goat, IRDye 800CW goat anti-rabbit and IRDye 680 goat anti-mouse).

### γH2AX flow cytometry analysis

CLL samples (1 × 10^6^ cells) were fixed in 70% ethanol and maintained at −20°C for at least 1 h. Next, cells were washed twice with cold PBS, and rehydrated for 10 min at 4°C in PBS containing 4% FBS and 0.1% Triton. Cells were resuspended in 100 μl of the same buffer with a 1/200 dilution of Alexa Fluor 488 Mouse anti-H2AX(pS139) antibody or Alexa Fluor 488 IgG1 k isotype control (both from BD Biosciences), incubated for 1 h at room temperature in darkness, washed and resuspended in PBS. Labeled cells were analyzed with FACSVerse flow cytometer (BD Biosciences).

### dCK assay

dCK activity was measured by a radiochemical assay using 50 μg of cellular protein as described in [14], with 10 μM [5-^3^H]-deoxycytidine (∼1000 cpm/pmol) and 5 mM Mg-ATP as substrates. Activities were expressed as fold change.

### Cytotoxicity and apoptosis assays

To assess the effect of ATR inhibitors on the sensitivity of CLL cells to UV-C or purine analogs, CLL cells (2 × 10^6^ cells /well) were incubated in 96-well plates during 24 h (for UV-C) or 48 or 96 h (for purine analogs) as indicated in figure legends. Cell viability was measured using the MTT assay, as previously detailed [66]. Each condition was done in triplicate in the same experiment. Caspase-3 activity was determined by a fluorometric assay with Ac-DEVD-AMC as substrate, as described in [41]. The cleaved form of procaspase-3 was analyzed by Western blot.

### DNA repair synthesis

DNA repair synthesis was analyzed by measuring incorporation of [^3^H-methyl]-thymidine into DNA, using the Millipore Multiscreen Assay System (Millipore, Billerica, USA) as previously described [43]. The assay was performed in the presence of 1 mM hydroxyurea in order to inhibit any residual semi-conservative DNA synthesis.

### Quantitative real-time polymerase chain reaction (qPCR)

Total cellular RNA was prepared using TRIzol (Invitrogen). Two μg of RNA was reverse transcribed with random hexamers using the RevertAid H Minus Reverse Transcriptase (Thermofisher Scientific) according to the manufacturer's protocols. The cDNA was subsequently amplified by qPCR using the KAPA SYBR FAST qPCR Kit (Kapa Biosystems, MA, USA) on a CFX96 Touch Real-Time PCR detection system (Bio-Rad). All reactions were performed in duplicate. PCR product specificity and genomic DNA contamination were verified by melting curve analysis and agarose gel electrophoresis. The relative quantification of gene expression was performed using the 2^−ΔΔCT^ method [67]. Primers used for amplification: PUMA forward (GACGACCTCAACGCACAGTA), PUMA reverse (GGAGTCCCATGATGAGATTGTA), BAX forward (CTGACGGCAACTTCAACTGG), BAX reverse (GTCCAGCCCATGATGGTTCT), Bcl-2 forward (GCACACCTGGATCCAGGATA), Bcl-2 reverse (CAGAGACAGCCAGGAGAAATC). TATA sequence binding protein forward, β-2-microglobulin and GAPDH were used as endogenous control to normalize expression within each sample.

### Statistical analysis

Results are expressed as the means ± SEM of at least three independent experiments. Statistical analysis was performed with GraphPad Prism 7.0 software using the one- or two-way ANOVA, followed by the Tukey's *post hoc* test. Changes were considered significantly different at *P* values < 0.05.

## SUPPLEMENTARY MATERIALS FIGURES AND TABLES



## Abbreviations

ATR: ataxia telangiectasia and Rad3-related
ATM: ataxia telangiectasia mutated
PI3K: phosphoinositide 3-kinase
CLL: chronic lymphocytic leukemia
CdA: 2-chloro-2′-deoxyadenosine (cladribine)
F-Ara-A: 2-flu oro-arabinosyladenine (fludarabine)
dCK: deoxycytidine kinase
DDR: DNA damage response
IR: ionizing radiation
UV-C: ultraviolet C
NER: nucleotide excision repair
PBMCs: peripheral blood mononuclear cells
γH2AX: H2AX phosphorylated at Ser-139
