# Supplementary material for: Reevaluation of ATR signaling in primary resting chronic lymphocytic leukemia cells: evidence for pro-survival or pro-apoptotic function
Source: Oncotarget. 2017 May 24;8(34):56906–20. doi: 10.18632/oncotarget.18144 (PMC5593612; doi:10.18632/oncotarget.18144)
Supplement: Supplementary file 1 [file oncotarget-08-56906-s001.pdf]

Reevaluation of ATR signaling in primary resting chronic lymphocytic leukemia cells: evidence for pro-survival or pro-apoptotic function

SUPPLEMENTARY MATERIALS

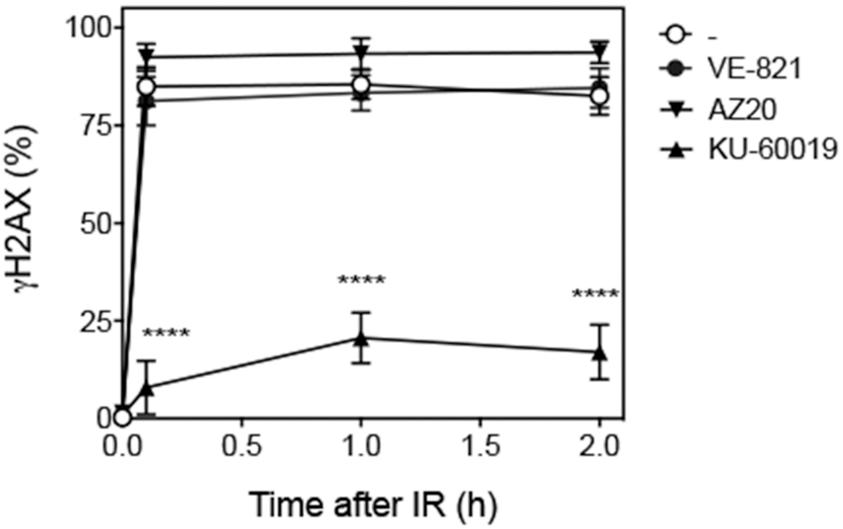

Supplementary Figure 1: Effect of ATR and ATM inhibitors on γH2AX accumulation induced by IR. CLL cells were preincubated with or without 10 µM VE-821, 1 µM AZ20 or 10 µM KU-60019 before IR (5 Gy). Phosphorylation of H2AX (γH2AX) was measured by flow cytometry at the indicated times, and expressed as fold increase over untreated cells. Results are means ± SEM of 3 independent experiments. Significance relative to the absence of inhibitors: ****P < 0.0001.

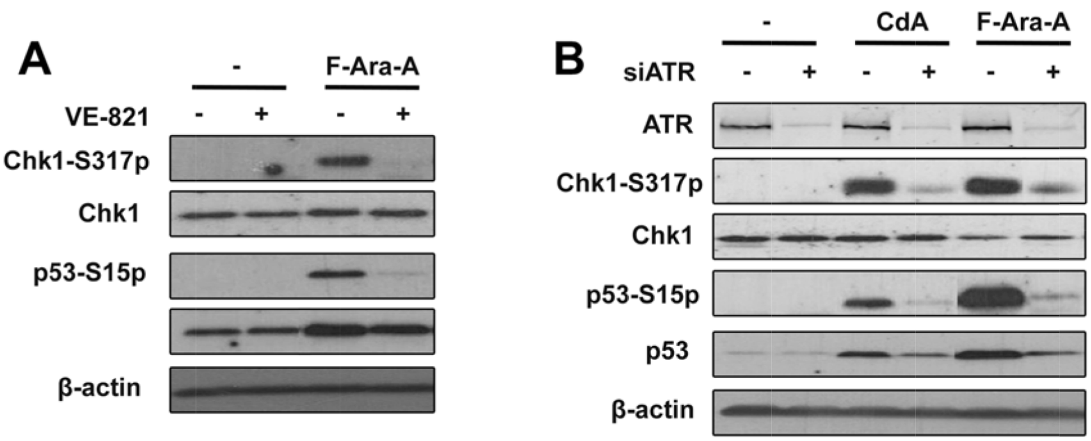

Supplementary Figure 2: Influence of the ATR inhibitor VE-821 or ATR siRNA on the induction of p53 by purine analogs in the CLL cell line EHEB. EHEB cells were preincubated with 10 µM VE-821 **(A)** or transfected for 48 h with scramble or ATR siRNA **(B)** before addition of 30 µM fludarabine (F-Ara-A) or 10 µM cladribine (CdA). Activation of p53 (p53-pSer15 and p53 protein level) was analyzed after 24 h of incubation. Phosphorylation of Chk1 at Ser-317 was measured to verify ATR inhibition. β-actin was used as loading control. It should be noted that high doses of fludarabine or cladribine were used in these experiments due to relatively low sensitivity of EHEB cells to purine analogs. EHEB cells were transfected with ATR siRNA using the Amaxa™ nucleofector and nucleofection Kit V (Amaxa, Cologne, Germany) as previously reported [18].

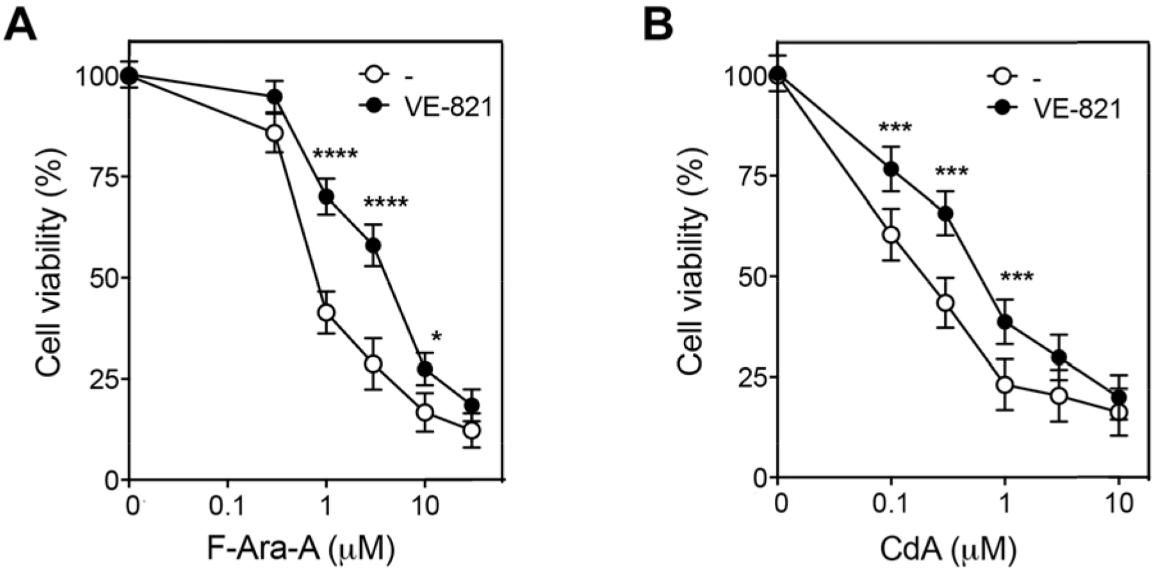

Supplementary Figure 3: Influence of VE-821 on purine analog-induced cytotoxicity. Primary resting CLL cells were preincubated with or without 10 µM VE-821 before addition of F-Ara-A **(A)** or CdA **(B)** at increasing concentrations. Cell viability was measured after 96 h by the MTT assay. Results are means ± SEM of 14 separate experiments. Significance relative to the absence of VE-821: *$P < 0.05$; ***$P < 0.001$; ****$P < 0.0001$.

Supplementary Table 1: Influence of VE-821 on the conversion of CdA and F-Ara-A into their triphosphate form and their incorporation into DNA

	Analog triphosphate (fold increase)		Analog into nucleic acids (fold increase)	
**Treatment**	**-**	**+ VE-821**	**-**	**+ VE-821**
**CdA (24h)**	1 ± 0.33	0.93 ± 0.06^{ns}	1 ± 0.17	0.94 ± 0.17^{ns}
**F-Ara-A (24h)**	1 ± 0.41	0.69 ± 0.15^{ns}	1 ± 0.16	0.71 ± 0.08*
**F-Ara-A (48h)**	1 ± 0.38	1.03 ± 0.11^{ns}	1 ± 0.34	0.96 ± 0.07^{ns}

CLL cells were preincubated in the presence or absence of 10 µM VE-821 before addition of labeled CdA (1 µM) or F-Ara-A (3 µM). F-Ara-ATP and CdATP, or their incorporation into nucleic acids were measured after 24 and/or 48 h of incubation as described previously [41]. Results are means ± SEM of 3 separate experiments. Significance relative to the absence of VE-821: * $P < 0.05$; ns: not significant. Concentrations of analog triphosphate in the absence of VE-821 were 33.1 ± 6.7 pmol/mg protein after 24 h of incubation with CdA, and 67.8 ± 5.2 and 71.3 ± 4.2 pmol/mg protein after 24 or 48 h of incubation with F-Ara-A. Incorporation into nucleic acids in the same conditions were 8.8 ± 1.5 pmol/mg protein after 24 h of incubation with CdA, and 1.2 ± 0.2 and 1.6 ± 0.4 pmol/mg protein after 24 or 48 h of incubation with F-Ara-A.

Supplementary Table 2: Clinical and biological characteristics of primary CLL samples

Patient ID no.	Gender	Age (year)	Stage	Prior therapy	Cytogenetic aberrations	IgVH mutational status
1	M	82	A	Ch	del13q	Unknown
2	M	51	A	None	None	Mutated
3	M	78	B	Ch	del13q	Unknown
4	M	73	A	None	del13q	Unknown
5	M	77	B	Ch	Trisomy 12	Unmutated
6	M	76	C	FCR; Ib; Id	None	Mutated
7	M	77	A	None	del13q	Unknown
8	F	75	C	None	Trisomy12	Unknown
9	F	68	A	None	del13q	Mutated
10	F	53	B	None	del13q	Mutated
11	M	45	A	None	del13q	Unknown
12	M	74	C	FCR; Ib	del13q, del17p	Unknown
13	M	66	A	None	del13q	Unkown
14	F	83	A	None	del13q	Unknown
15	M	56	A	ChOP; FCR	del13q	Unknown
16	F	91	A	Ch	del13q	Unknown
17	F	66	A	None	None	Unknown
18	M	59	C	FCR; O; D	del13q	Unmutated
19	F	85	B	Ch	None	Unknown
20	F	65	A	B; R	del13q	Mutated

(*Continued*)

Patient ID no.	Gender	Age (year)	Stage	Prior therapy	Cytogenetic aberrations	IgVH mutational status
21	M	79	A	None	del13q	Unknown
22	F	57	A	None	del13q	Mutated
23	M	73	A	Ch; Dexa-Beam; Z; Ib; IdR	del13q; del17p	Unknown
24	M	47	B	FCO	None	Unmutated
25	F	78	A	None	None	Mutated
26	M	68	B	None	Trisomy 12, del13q	Mutated
27	F	68	A	None	Unknown	Unknown
28	M	68	A	None	del13q	Unknown
29	M	68	A	None	del13q	Mutated
30	M	56	B	None	del13q	Unknown
31	M	58	B	FCO	Trisomy12; del 17p	Unmutated
32	M	81	A	None	Unknown	Unknown
33	F	65	B	ACP-196; Ib	del13q	Mutated
34	F	66	A	None	Trisomy 12	Unknown
35	F	78	A	None	Unknown	Unknown
36	M	61	A	FCR; V	del13q	Undeterminable
37	M	78	A	None	Unknown	Unknown
38	F	58	A	None	del13q	Mutated
39	M	80	C	FCR	del13q	Mutated
40	F	55	A	None	del13q; del17p	Mutated

M: Male; F: Female; Ch: Chlorambucil; FCR: fludarabine/cyclophosphamid/rituximab; Ib: ibrutinib; Id: idelalisib; O: ofatonumab; D: duvelisib; B: bendamustine; Z: anti CD37; IdR: idelalisib/rituximab; FCO: fludarabine/cyclophosphamid/ofatonumab; V: venetoclax.
